# Genitourinary and Syphilitic Diseases

**Published:** 1903-10

**Authors:** Byron H. Daggett

**Affiliations:** Buffalo, N. Y.


### PROGRESS IN MEDICAL SCIENCE.

### Genitourinary and Syphilitic Diseases.

Conducted by BYRON H. DAGGETT, M. D., Buffalo, N. Y.

### ALBUMOSURIA.

C. R. Burr (before the Portland, Me., Medical Club, February 5, 1903,) stated that albumosuria signifies albumoses in the urine. It is a pathological state, usually unappreciated. It is frequently confounded with peptonuria. The dividing line is very artificial. Many cases of albumosuria have been described as peptonuria, although deutroalbumose was present. This substance closely resembles peptone, hence the error.

Albumoses are intermediate products of digestion, four in number, to-wit: proto-, hetero-, dys-, and deutroalbumose. One is changed to another by hydrolic cleavage of absorption of water. Deutroalbumose absorbs water and becomes peptone, peptone absorbs water and splits up into the waste products of digestion, leucin, tyrosin, and the like. This hydrolysis is effected by the ferments or enzymes. The ferments are supplied by glands and bacteria. There have been described fifty varities of intestinal bacteria. Some of these ferments form sugar from starch, some peptone from proteid and others disintegrate fat. If these products are not absorbed disintegration changes sugar into lactic acid and alcohol; peptone into amido-acid; leucin and tyrosin into skatol, indol and phenol; fats into voleric and butyric acids.

Peptone is the final product of proteid digestion and as such is absorbed. Peptones and albumoses are found in the chyme, but not in normal blood. Serum albumin takes their place in that fluid. Peptones are absorbed by epithelial cells and leucocytes, which dehydrate and change them into albumin.

Jones and Hunt, in Gould's American Year Book of Medicine and Surgery for 1902, propose a law which might be called the reversible zymolysis, to-wit: the continual removal of one of the products of a chemic reaction causes its continual formation, also that the accumulation of these products induce retarded reaction.

Hill (*Am. Chem. Soc. Jour.*, LXXIII.,) says that the enzmye will decompose or change maltose in dextrose and dextrose into maltose until a certain condition of equilibrium is established, that if one is removed the other is formed at the expense of the one present until equilibrium is established. The fat splitting ferment, lipose, will change ethyl-butyrate into its corresponding acid and alcohol, and out of these reconstruct ethyl-butyrate. Albumoses are not absorbed into the blood under normal conditions. Albumoses once in the circulation tend to remain until further digested. They appear in the urine as deutro-albumose and peptone. Proto-albumose and hetero-albumose in the blood are excreted as deutro-albumose, and deutro-albumose as peptone.

Gillespie believes that albumosuria is due to the excessive production and destruction of leucocytes. Clinically, albumosuria is found where there is leucocytosis, and peptonuria where there is pus. The leucocyte has its own digestion ferment and can produce albumoses.

In leucocytosis there is a disturbance of the osmatic relations of the blood elements. The chlorids go to the cells and the albumins and phosphates to the serum. It is probable that a large part of the albumoses present in the blood of febrile cases is derived in this manner from the leucocytes. Dead leucocytes are digested, assisted by the ferments of pyogenic bacteria.

Pus contains serum, albumin, albumoses, peptones, leucin, tyrosin, cholesterin, and the like.

### DETECTION.

Clinically, it is not necessary to distinguish between the different forms of albumoses present in the urine.

For their detection I prefer the methods given by Ogden, which are as follows:

1. Take a small portion of urine in a test tube, and warm gently. A precipitate appears which is redissolved on boiling and reappears on cooling.

2. Acidulate the urine with acetic acid, and add a few drops of a saturated solution of sodium chloride. A precipitate forms which disappears on heating and reappears on cooling.

3. Add a few drops of nitric acid to the urine in a test tube. If the acid is not in excess a precipitate is formed which disappears on boiling and reappears on cooling.

4. Add acetic acid, avoiding an excess, and then add a few drops of a solution of potassium ferro-cyanide (1.10). A precipitate is formed which disappears on boiling and reappears on cooling.

5. Completely saturate the urine at boiling point with neutral ammonium sulphate; filter and wash the precipitate with saturated solution of ammonium sulphate. Dissolve the precipitate in water or dilute sodium chloride solution and apply the beuret test. If albumoses are present a rose color results.

The beuret test is commonly applied in cases of peptonuria. It is unreliable because not only peptones, but albumoses and urobilin respond to it as well.

The only sure test is to proceed as in No. 5; that is, to saturate the urine (which should be acid in reaction) with ammonium sulphate, filter out the precipitate, and add to the filtrate either potassio-mercuric iodide or picric acid. If a precipitate appears it is peptone.

